# Toward system-level integration of organoids for regenerative medicine

**DOI:** 10.1093/burnst/tkag031

**Published:** 2026-04-16

**Authors:** Chuqing Zhou, Yuanli Ye, Jinrui Cai, Mingxuan Li, Yuchun Tang, Qiaoli Xie, Xiao Xiang, Mingxing Lei

**Affiliations:** Key Laboratory of Biorheological Science and Technology of Ministry of Education and 111 Project Laboratory of Biomechanics and Tissue Repair, College of Bioengineering, Chongqing University, No. 174 Shazheng Street, Shapingba District, Chongqing, Chongqing 400044, China; Key Laboratory of Biorheological Science and Technology of Ministry of Education and 111 Project Laboratory of Biomechanics and Tissue Repair, College of Bioengineering, Chongqing University, No. 174 Shazheng Street, Shapingba District, Chongqing, Chongqing 400044, China; Key Laboratory of Biorheological Science and Technology of Ministry of Education and 111 Project Laboratory of Biomechanics and Tissue Repair, College of Bioengineering, Chongqing University, No. 174 Shazheng Street, Shapingba District, Chongqing, Chongqing 400044, China; Key Laboratory of Biorheological Science and Technology of Ministry of Education and 111 Project Laboratory of Biomechanics and Tissue Repair, College of Bioengineering, Chongqing University, No. 174 Shazheng Street, Shapingba District, Chongqing, Chongqing 400044, China; Key Laboratory of Biorheological Science and Technology of Ministry of Education and 111 Project Laboratory of Biomechanics and Tissue Repair, College of Bioengineering, Chongqing University, No. 174 Shazheng Street, Shapingba District, Chongqing, Chongqing 400044, China; Key Laboratory of Biorheological Science and Technology of Ministry of Education and 111 Project Laboratory of Biomechanics and Tissue Repair, College of Bioengineering, Chongqing University, No. 174 Shazheng Street, Shapingba District, Chongqing, Chongqing 400044, China; Key Laboratory of Biorheological Science and Technology of Ministry of Education and 111 Project Laboratory of Biomechanics and Tissue Repair, College of Bioengineering, Chongqing University, No. 174 Shazheng Street, Shapingba District, Chongqing, Chongqing 400044, China; Key Laboratory of Biorheological Science and Technology of Ministry of Education and 111 Project Laboratory of Biomechanics and Tissue Repair, College of Bioengineering, Chongqing University, No. 174 Shazheng Street, Shapingba District, Chongqing, Chongqing 400044, China

**Keywords:** Organoids, Integrated organoids, Regenerative medicine, Tissue repair, Vascularized, Innervated, Immune-competent

## Abstract

Regenerating complex human tissues requires proper cellular assembly and the recapitulation of coordinated functions across multiple biological levels. Organoids, as self-organized three-dimensional cellular structures, provide powerful models for rebuilding organ architecture and studying developmental processes. However, their regenerative potential remains limited by the lack of vascular, neural, and immune integration, which are essential for tissue development, homeostasis, and repair. Recent studies indicate that the progression from tissue-level organization to organ-level coordination and ultimately to system-level functionality depends on dynamic intercellular communication, feedback signaling, and niche interactions. The integration of biochemical, biomechanical, and bioelectrical cues enables multicellular systems to achieve synchronized growth, patterning, and functional adaptation. Complementary bioengineering strategies further guide these intrinsic processes by modulating spatial organization, microenvironmental signals, and intercellular connectivity. This review summarizes emerging methodologies and molecular mechanisms underlying system-level integration in organoids and discusses how these biological processes may bridge the gap between *in vitro* morphogenesis and *in vivo* functional regeneration.

Highlights

- Organoids integrate vascular, neural, and immune components for system-level functionality.
- Vascular networks improve survival, maturation, and host tissue integration.
- Neural circuits enable functional patterning and organ-specific responses.
- Immune competence supports tissue homeostasis, remodeling, and regenerative potential.
- System-level integration drives organoids toward fully functional, clinically translatable regenerative therapies.

## Background

The regeneration of complex and functional human tissues remains one of the foremost challenges in regenerative medicine. Advances in stem cell biology have provided the foundational tools to generate tissues *in vitro*; yet, a substantial gap persists between constructing cellular assemblies and achieving durable, functional repair *in vivo*. Organoids, self-organizing three-dimensional (3D) cellular structures that recapitulate essential aspects of organ-specific architecture and physiology, have emerged as transformative models for studying organ development, disease mechanisms, and regeneration [1–5]. These organoids can be derived from embryonic stem cells, adult stem cells (ASCs), or induced pluripotent stem cells (iPSCs) and are generated by guiding cells through developmental cues and self-organization processes [6–8]. In practice, this involves providing cells with appropriate biochemical signals, extracellular matrix environments, and culture conditions, allowing them to spontaneously assemble into miniaturized, organ-like structures that capture essential features of the target tissue [9, 10].

**Figure 1:**
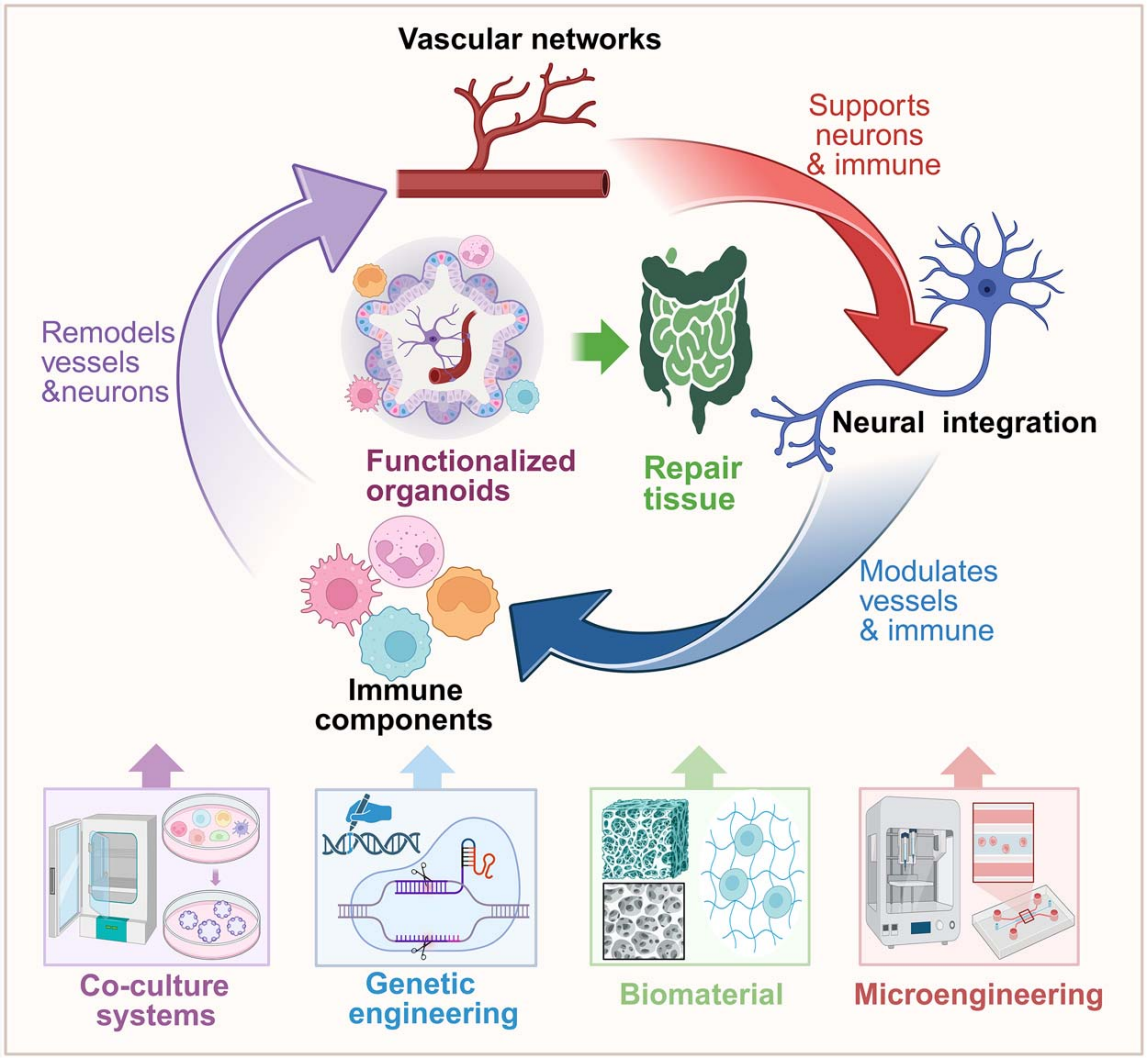
Conceptual framework for system-level integration for regenerative medicine. Organoids can be engineered to achieve functional, system-level integration incorporating vascular, neural, or immune components through emerging methodologies, such as co-culture systems, genetic engineering, biomaterials, and microengineering strategies, to enhance physiological regulation, tissue remodeling, and host communication

Despite their promise, most organoids often resemble fetal tissues, exhibiting limited survival, maturation, and functional integration with host tissue after transplantation [11, 12]. Challenges come from the absence of critical physiological systems, including vascular networks that supply oxygen and nutrients, neural integration that enables functional regulation [13, 14], and immune competence that supports tissue remodeling and graft-host communication [15]. Moreover, the functional contribution of each system also varies by organ type, adding to the existing challenge of achieving fully functional organs. Thus, addressing these gaps is therefore fundamental for realizing clinically meaningful organoid-based therapies (Fig. 1).

The progression from tissue-level organization to organ-level coordination and ultimately to system-level functionality depends on dynamic intercellular communication, feedback signaling, and the local niche. The integration of biochemical, biomechanical, and bioelectrical cues enables multicellular systems to achieve synchronized growth, spatial patterning, and functional adaptation. For example, in our established skin organoids, interactions among epidermal, dermal, and melanocyte progenitor cells drive the formation of hair follicles and pigmentation patterns [10]. In addition, incorporating vascular components not only supports nutrient delivery but also enables reciprocal crosstalk between endothelial cells and dermal papilla, thereby promoting both hair regeneration and angiogenesis [16]. Self-organization in skin organoids proceeds through sequential phases, from dissociated cells to aggregates, polarized cysts, coalesced planar tissue, and ultimately fully patterned organoids [17]. These transitions are guided by coordinated mechano-chemical signaling, which regulates tissue fluidity, cell movements, and matrix remodeling. This process underlies tissue morphogenesis and ensures functional maturation, linking tissue-level patterning to organ- and system-level functionality [9]. Such findings highlight the potential to further integrate vascular, neural, and immune components, advancing organoids toward fully functional, system-level regenerative models.

Recent advances in bioengineering have begun to overcome the limitations of conventional organoids by emphasizing vascularization, innervation, and immune competence as a critical triad for unlocking regenerative potential. Complementary strategies, such as biomaterial design, spatiotemporal co-culture, and controlled fusion of distinct organoid types, are now being used to integrate these essential components and generate organoids with higher-order complexity. These systems have demonstrated enhanced graft survival, accelerated maturation, and improved host integration across diverse organoid models. This review critically summarizes emerging methodologies and molecular mechanisms underlying the incorporation of vascular, neural, and immune modules, highlighting how these modules and their impact on organoid performance. We propose a conceptual framework for next-generation organoids that move beyond structural mimicry toward functional integration and system-level regenerative capability.

## Review

### Vascularization as a critical determinant of organoid survival and integration

#### The challenge of graft ischemia

The paramount challenge for organoid-based regeneration is graft ischemia. Without intrinsic vascular networks, transplanted organoids depend entirely on passive diffusion from the host environment, which is highly inefficient. When organoid thickness exceeds ~200 μm, oxygen and nutrient diffusion become insufficient to sustain the inner regions, leading to central necrosis and severely limiting long-term viability and functional maintenance [18–20]. Beyond survival, the absence of functional vasculature disconnects the graft from systemic circulation, preventing participation in key physiological processes such as endocrine signaling, immune surveillance, and drug metabolism [21, 22]. As a result, these grafts remain isolated, non-functional “islands” within host tissues.

#### Methodological approaches to establish perfusable vascular networks

**Figure 2:**
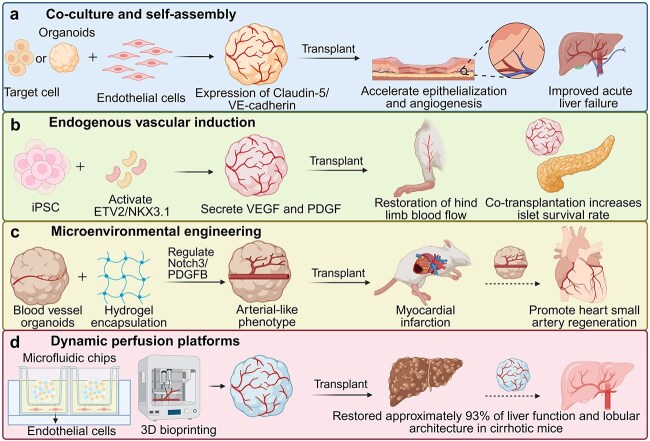
Overview of approaches for constructing vascularized organoids. (**a**) Co-culture and self-assembly: target cells or organoids are co-cultured with endothelial cells to induce spontaneous vascular network formation. Direct cell–cell interactions enhance endothelial barrier integrity and vascular stability by upregulating key junctional proteins such as Claudin-5 and VE-cadherin. (**b**) Endogenous vascular induction: ectopic activation of transcription factors (e.g. ETV2, NKX3.1) in iPSCs drives the secretion of proangiogenic factors including VEGF and PDGF, promoting vasculogenesis and pericyte recruitment. (**c**) Microenvironmental engineering: cell encapsulation within hydrogels regulates signaling pathways (e.g. Notch3, PDGFB) through biochemical and mechanical modulation of the biological matrix, guiding engineered vasculature toward a mature, artery-like phenotype. (**d**) Dynamic perfusion platforms: microfluidic chips and 3D bioprinting enable precise spatial organization of endothelial and target cells, supporting the formation of scalable, perfusable, and highly biomimetic organoids. *iPSCs* induced pluripotent stem cells, *VEGF* vascular endothelial growth factor, *PDGF* platelet-derived growth factor, *ETV2* ETS variant transcription factor 2, *NKX3.1* NK3 homeobox 1

Co-culture and self-assembly: one fundamental strategy is the co-culture of organoid progenitors with endothelial and supporting cells within a 3D matrix. This method harnesses the intrinsic ability of these cells to self-organize into nascent vascular networks. For example, brain organoids co-cultured with human brain microvascular endothelial cells formed vascular structures expressing tight junction proteins (Claudin-5 and VE-cadherin) and basement membrane components (Collagen IV and Laminin α5), reducing internal apoptosis and improved overall viability [23]. Similarly, in liver organoids, the work of Saiki *et al.* and Zhu *et al.* complements each other. The former co-cultured iPSC-derived hepatic sinusoidal endothelial progenitor cells with hepatic bud organoids to construct human hepatic bud organoids possessing functional sinusoidal vascular networks (Fig. 2a). The latter employed a co-culture system of human ASC-derived liver organoids and iPSC-derived vascular organoids (VOs) to form vascularized liver micro-tissues, enhancing the maturity of hepatocytes and their albumin secretion function *in vitro* [24, 25]. These examples illustrate that multicellular interactions can guide vascular patterning and maturation within organoids.

Endogenous vascular induction: complementary to co-culture, endogenous induction approaches use developmental cues to drive vascular differentiation within organoids. Activation of transcription factors such as ETS variant transcription factor 2 (ETV2) and NK3 homeobox 1 (NKX3.1) induces progenitors to co-differentiate into endothelial and mural cells, forming patent vascular networks in a few days (Fig. 2b) [26, 27]. Table 1 summarizes the transcription factor-based approaches for generating endothelial cells in organoid systems [28–37]. In brain organoids, such strategies not only recapitulated up to 80% of human fetal brain cell types but, more importantly, revealed that endothelial cells actively secrete a variety of signaling molecules, including vascular endothelial growth factor (VEGF), bone morphogenetic protein (BMP), platelet-derived growth factor (PDGF), and insulin-like growth factor (IGF), to regulate neurodevelopmental processes. This secretion plays a critical role in maintaining intermediate progenitor cells in the hindbrain (Fig. 2b) [38]. These findings highlight that vasculature contributes actively to tissue patterning and organoid maturation, beyond merely providing nutrient support.

**Table 1:** Transcription factors and upstream pathways applied for vascular induction in organoids

Category	Factor/pathway	Mechanism	Refs.
Upstream signaling regulators	BMP4 + CHIR99021; growth factors (e.g. FGF2, VEGF)	Activate mesoderm and vascular progenitor fate, indirectly activating endothelial transcription factors.	[28–31]
Core TF module	ETV2	Triggers EC differentiation from fibroblasts or pluripotent stem cells without relying on traditional growth factors.	[32–34]
Cooperative TF modules	ETV2 + SOX17	Enhance endothelial reprogramming efficiency and vascular maturation, promoting functional vessel formation *in vivo*.	[35]
	ETV2 + NKX3.1	Drive endothelial and mural cell co-differentiation and enable formation of functional vascular networks.	[27, 36]
Cross-lineage TF modules	NEUROD1; MYOD1 + BAF60C	Induced overexpression in developing neuro-vascular and myo- VOs.	[37]

*VEGF* vascular endothelial growth factor, *ETV2* ETS variant transcription factor 2, *NKX3.1* NK3 homeobox 1, *FGF2* fibroblast growth factor 2, *EC* endothelial cell, *VOs* vascular organoids

Microenvironmental engineering: vascular maturation is further influenced by the surrounding microenvironment. Engineered biomaterials critically regulate organoid development by delivering precisely tunable biophysical and biochemical cues beyond what conventional culture systems can provide [39, 40]. For instance, norbornene-modified hyaluronic acid hydrogels demonstrate that intermediate stiffness (551 Pa) enhances cluster of differentiation 31 (CD31) expression and yes-associated protein/transcriptional coactivator with PDZ-binding motif (YAP/TAZ) activation, promoting endothelial maturation, whereas more compliant matrices (190 Pa) favor vascular connectivity and branching, highlighting a tradeoff between mechanotransduction and network formation [41]. Viscoelastic hydrogels support arteriole-like differentiation and smooth muscle incorporation via Notch3 and PDGFB signaling (Fig. 2c) [42]. In parallel, extracellular matrix-based materials such as Matrigel provide a permissive biochemical microenvironment enriched in basement membrane proteins and growth factors, supporting stem and progenitor cell self-organization, proliferation, and differentiation [43]. Adhesive ligands and growth factor-binding sites in these matrices further regulate endothelial adhesion, migration, and angiogenic signaling. For example, exposure of adipose-derived stem cells to a gelatin sponge rich in arginine-glycine-aspartate (RGD) motifs triggered a potent pro-angiogenic and pro-inflammatory cascade via α5β1 and αvβ3 integrins, leading to significant upregulation of interleukin-6 (IL-6), IL-8, VEGF, and angiopoietin-1. Conditioned medium from these cells markedly enhanced the formation of CD31-positive tubular structures in human embryonic stem cell-derived VOs, and this effect was almost entirely abrogated by the RGD antagonist cilengitide [44]. Such mechanistically guided biomaterial design provides a framework for supporting robust vascular network formation in organoid systems, which can be further adapted to integrate neural and immune components for regenerative medicine applications.

Dynamic perfusion platforms: in addition to static culture, dynamic perfusion methodologies such as microfluidic chips and 3D-printed platforms can simulate physiological flow conditions, promoting network maturation, and organoid viability (Fig. 2d) [45]. For example, placental organoids were generated from human trophoblast stem cells and cultured within a microphysiological system incorporating a vascular endothelium under dynamic perfusion conditions. This setup promoted in situ differentiation, long-term viability, and maturation of trophoblasts, while co-culture with endothelial cells activated innate immune pathways and enhanced the secretion of antiviral and trophoblast-specific factors, demonstrating that dynamic vascular environments support complex tissue functionality [46]. 3D bioprinting enables the predefine of vascular tree architectures, further enabling precise spatial organization and functional integration [47, 48]. These approaches highlight that mimicking physiological flow and spatial cues is crucial for achieving fully functional, vascularized organoids.

#### Regenerative medicine applications and evidence

Vascularized organoids have demonstrated direct applications in regenerative medicine, particularly in wound and soft tissue repair. In skin regeneration, co-culturing endothelial cells with mesenchymal stem cells to 3D spheroids, followed by delivery via a hydrogel carrier, markedly enhances post-transplantation angiogenesis, resulting in a 31% increase in epidermal layer recovery rate (Fig. 2a) [49]. This pre-vascularization approach not only mitigates nutrient limitations but also establishes a supportive microenvironment for integration with host tissues, including nerves and muscles [50]. In refractory wound models, such as diabetic wounds, these engineered organoids have shown significant promotion of epithelialization and angiogenesis, substantially improving wound closure rates [51]. Mechanistically, neonatal mouse-derived organoids exhibit higher expression of cardiotrophin-1 (CTF1) and interleukin-6 signal transducer (IL6ST) compared with adult mouse-derived organoids. Administration of IL6ST or α-linolenic acid promotes elongation and tight arrangement of endothelial cells, upregulates Fms-related receptor tyrosine kinase 4 (FLT4), and enhances self-organization, indicating that both factors regulate angiogenesis. Further transplantation experiments revealed that activation of IL6ST signaling significantly increases CD31-positive endothelial cells, proliferating cell nuclear antigen-positive proliferating endothelial cells, and FLT4 expression in engrafted skin, revealing how specific molecular pathways coordinate vascular network formation [16]. Vascularized environments also influence the differentiation of cartilage organoids, supporting the formation and integration of functional bone-cartilage units. In double-layer cartilage organoid/gelatin methacryloyl (GelMA) composites, gradient vascular conditions have been shown to direct lineage-specific differentiation, with the avascular upper layer favoring cartilage formation and the vascular-rich lower layer promoting subchondral bone development. In a rabbit articular defect model, 12 weeks after implantation of VEGF-treated cartilage organoid composites, the defect area integrated well with native tissue. Cartilage-specific extracellular matrix components were enriched, illustrating how vascular cues coordinate spatially distinct tissue maturation and cross-layer matrix organization [52]. These findings collectively highlight that effective vascularization enhances organoid survival, integration, and functional restoration, emphasizing its translational potential in trauma and wound repair.

The successful implementation of vascularization strategies has extended these findings across multiple organ systems. In the liver, vascularized organoids generated via co-culture with iPSC-derived endothelial cells have been transplanted into mouse models of acute liver failure and hemophilia A, where they integrated with host circulation and significantly ameliorated disease pathologies (Fig. 2a) [24, 25]. Similarly, ETV2- and NKX3.1-induced VOs restored perfusion in murine hindlimb ischemia and, when co-transplanted with pancreatic islets, enhanced graft survival, and insulin secretion, highlighting molecularly mediated angiogenic support for diabetes therapy (Fig. 2b) [26, 27]. In cardiac regeneration, organoids preconditioned in viscoelastic hydrogels and transplanted into myocardial infarction models promoted host arteriole regeneration and improved cardiac function, underscoring the importance of vascular maturation in functional tissue recovery (Fig. 2c) [42]. In neurodegenerative disease research, the integration of microglia and a vascular system into an Alzheimer’s disease (AD) organoid model has enabled the recapitulation of hallmark pathologies. Upon exposure to brain extracts from patients with sporadic AD, these organoids rapidly developed Aβ plaques, Tau tangles, synaptic loss, and neuroinflammation [53]. Moreover, under controlled flow conditions, organ-on-chip devices integrated with biosensors demonstrated that flow-induced mechanical stimulation can synchronize the maturation of endothelial cells and hepatocytes. The integrated sensor chip also identified a critical turning point on Day 5, marked by peak biomarker levels, and defined an optimal transplantation window. Transplantation within this window restored ~93% of liver function and lobular structure in mice with liver cirrhosis, whereas grafts transplanted outside this window showed a 50%–70% loss of therapeutic efficacy (Fig. 2d) [54].

Beyond classical organ models, vascularized organoids have been adapted to investigate metabolic and developmental systems. For example, a vascularized adipose organoid model demonstrated a tight functional coupling between vasculature and adipose components. Compared with non-vascularized controls, these adipose organoids exhibited a healthier metabolic phenotype, more stable inflammatory responses, and the ability to respond synchronously to drugs like Celastrol, which simultaneously inhibited both angiogenesis and adipogenesis [55]. Vascularized human brain organoids combined with single-cell transcriptomic analysis precisely have precisely delineated the molecular responses of distinct cell types, including GABAergic neurons, astrocyte precursor cells, and microglia, to hypoxic stress. These studies further revealed the active role of vascular components in modulating inflammatory signaling and mediating neuroprotective mechanisms [56]. Collectively, recent advances in vascularized organoid research provide compelling evidence for their translational potential in regenerative medicine. These organoids not only enhance survival, integration, and physiological functionality in preclinical models but also elucidate key molecular mechanisms, such as growth factor signaling, matrix remodeling, and metabolic coupling, that underpin system-level regenerative outcomes across diverse tissues.

Beyond their structural and metabolic functions, vascular networks serve as dynamic hubs that integrate neural and immune signaling within regenerating tissues. For example, in multi-region brain organoids, endothelial cells form paracrine signaling networks with neural progenitors, supporting region-specific differentiation and axon development [38]. In parallel, the vasculature provides a primary conduit for immune signaling and trafficking, enabling spatiotemporal control of inflammation and tissue protection; flow-mediated shear stress further modulates endothelial immunogenicity; for example, in vascularized placental organoids, endothelial cells enhance trophoblast viability and activate innate immune pathways, including antiviral interferons, illustrating how a vascular niche modulates immune responses [46]. Thus, vascularization should not be viewed as an isolated engineering goal but rather as the foundational infrastructure that supports neuro-immune coordination. Given that functional tissues require neural control to translate vascular support into organ-level responsiveness, the next critical dimension of integration lies in establishing effective innervation.

### Innervation as the foundation of functional integration and organ control

#### The problem of a “dumb” graft

A major limitation in organoid and tissue transplantation lies in the creation of “dumb” grafts. Such grafts remain physiologically disconnected, unable to communicate with the host’s neural circuitry, and thus fail to achieve true functional restoration [57, 58]. For organoids to serve as surrogate organs, they must engage in reciprocal signaling with the host nervous system. Peripheral nerves regulate multiple aspects of regeneration, including stem cell differentiation, angiogenesis, and immune balance, through neurotrophic and neuropeptidergic signaling [59]. Thus, restoring neural control represents a central methodological goal for building functional regenerative systems.

#### Methodological strategies to establish neural integration

**Figure 3:**
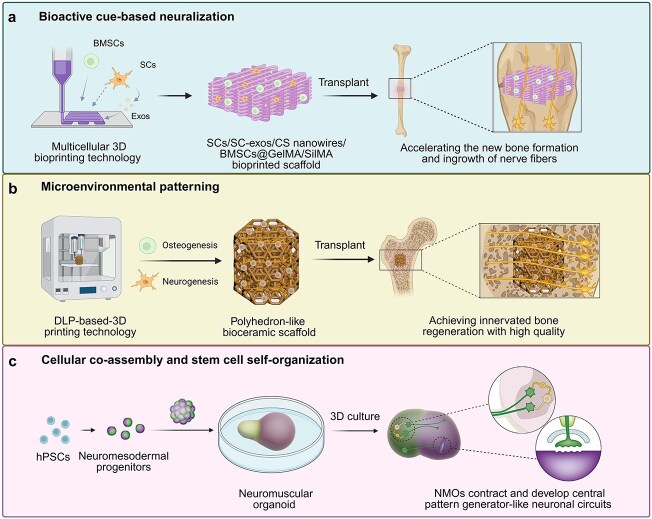
Integrated approaches for neuralized bone and neuromuscular regeneration. (**a**) Bioactive cue-based neuralization: multicellular 3D bioprinting of BMSCs, SCs/SC-Exos, and CS nanowires within GelMA/SiMA hydrogels synergistically activates osteogenic and neurogenic pathways for coordinated bone-nerve regeneration. (**b**) Microenvironmental patterning: DLP-printed polyhedral bioceramic scaffolds with biomimetic open pores guide SC alignment and promote innervated bone formation. Reproduced with permission [62]. Copyright 2023, Wiley-VCH. (**c**) Cellular co-assembly and stem cell self-organization: hPSC-derived spinal and skeletal muscle organoids self-assemble into NMOs that form functional motor units and exhibit rhythmic, central-pattern-generator-like neural activity. Reproduced with permission [63]. Copyright 2020, Cell Press. *BMSCs* bone marrow mesenchymal stem cells, *CS* calcium silicate, *SCs* schwann cells, *SC-exos* schwann cells-derived exosomes, *hPSC* human pluripotent stem cell, *NMOs* neuromuscular organoids

Bioactive cue-based neuralization: Controlled biochemical modulation represents one of the earliest and most tractable methodologies to promote innervation. Neural-inductive cues, such as neurotrophic factors, ion signaling, or exosome-mediated transfer, can be systematically integrated into culture systems to guide neurogenesis and neurovascular coupling. For example, incorporating calcium silicate nanowires into GelMA bioinks enables the controlled release of Ca^2+^ and SiO₃^2−^ ions, which activate Wingless-related integration site signaling pathway (Wnt)/β-catenin and MAPK/ERK pathways to promote osteogenesis, while concurrently stimulating TGF-β/Smad and PI3K-Akt signaling in Schwann cells (SCs) to enhance brain-derived neurotrophic factor and glial cell-derived neurotrophic factor expression [60]. In parallel, SC-derived exosomes (SC-exos) were integrated with bone marrow mesenchymal stem cells (BMSCs) in a hybrid GelMA/SilMA hydrogel bioink. SC-exos enhanced BMSC osteogenesis by regulating the TGF-β signaling pathway via the transfer of let-7c-5p, while promoting the migration and tube-formation capacity of endothelial progenitor cells. These coordinated effects reconstructed SC-mediated nerve–bone crosstalk *in vivo*, yielding dense NF200^+^ nerve fibers, improved vascularization, and neurovascularized bone regeneration (Fig. 3a) [61]. These molecularly defined models demonstrate how dynamic signaling modulation can reproduce the crosstalk between nerve and stromal compartments essential for functionally innervated regeneration.

Microenvironmental patterning: beyond soluble cues, spatial organization provides critical guidance for neuronal arrangement and tissue patterning. A digital light processing (DLP)-based 3D-printed β-TCP bioceramic scaffold with a tree-like architecture was designed as a 3D platform to support the co-culture of BMSCs and SCs. The scaffold’s leaf-like and gradient structures supported BMSC-SC adhesion, proliferation, and osteo-neurogenic differentiation. SC-derived NGF activated TrkA-Wnt/β-catenin signaling in BMSCs, while BMSCs enhanced neurotrophic factor secretion from SCs, establishing a reciprocal neuro-osteogenic niche. When implanted into rabbit femoral defects, the scaffolds markedly increased NF200^+^ nerve fiber density compared with conventional designs, achieving structurally and functionally innervated bone regeneration [64]. In another study, polyhedral and fiber-patterned scaffolds generated by advanced 3D printing techniques have been shown to enhance BMSCs proliferation, adhesion, and osteogenic differentiation through activating the PI3K-Akt pathway (Fig. 3b) [62]. Polycaprolactone microfibers with adjustable angles (30°-90°) fabricated via near-field electrostatic printing demonstrated that 90° fiber alignment significantly promoted osteogenesis in BMSCs by upregulating the miR-222–5p/cbfb/Runx2 pathway [65]. Such studies highlight the methodological significance of precisely controlled spatial patterning.

Cellular co-assembly and stem cell self-organization: advances in 3D co-culture and stem cell self-organization have expanded the toolkit for reconstructing functional neural circuits. This approach was first exemplified in intestinal organoids, where neural crest cells were integrated into developing human intestinal organoids to generate a functional enteric nervous system (ENS) [66]. Similar strategies have been extended to the neuromuscular system, where co-culturing human muscle progenitors with motor neurons within 3D hydrogels enables the spontaneous formation of functional neuromuscular junctions (NMJs). Motor neuron-secreted agrin activates the MuSK-rapsyn complex to cluster acetylcholine receptors (AChRs), while neuregulin-1 (NRG1)-ErbB signaling mediates the γ-to-ε AChR subunit switch, promoting synaptic maturation. The resulting NMJs display glutamate-evoked calcium transients and spontaneous endplate potentials, closely mimicking human neuromuscular function *in vitro* [67]. Moreover, human pluripotent stem cell (hPSC)-derived axial stem cells can give rise to spinal motor neurons and skeletal muscle cells that self-organize into neuromuscular organoids (NMOs) with long-term 3D stability. Driven by Wnt/fibroblast growth factor (FGF) signaling, these neuromesodermal progenitors establish posterior axial identity and co-develop into motor neurons, myofibers, and terminal SCs, which assemble functional NMJs marked by acetylcholine receptor clustering. The mature NMOs exhibit spontaneous rhythmic activity and central pattern generator-like circuits, faithfully recapitulating human neuromuscular network organization (Fig. 3c) [63]. hPSCs can be guided to form skin organoids containing hair follicles, sebaceous glands, Merkel cells, and sensory neurons. By modulating BMP, FGF, and Wnt signaling, ectodermal and neural crest-derived cells co-develop into layered epidermal-dermal structures, where epithelial-mesenchymal interactions drive hair follicle formation and sensory innervation, recapitulating the neurofunctional architecture of fetal human skin [50]. Methodologically, these models combine defined progenitor populations (e.g. neural crest cells, motor neurons, or SCs) with developing organoids to achieve self-patterned innervation.

#### Regenerative medicine applications and evidence

Among various regenerative applications, the neuralization of bone and muscle organoids has advanced most prominently, providing compelling preclinical evidence. The co-delivery of propranolol and calcitonin gene-related peptide (CGRP) synergized with silicon ions to promote tube formation in human umbilical vein endothelial cells (HUVECs) and upregulate osteogenic genes in BMSCs. After 10 weeks of implantation into rat cranial defects, bone volume/total volume (BV/TV) reached 37.56 ± 0.91%, indicating that a higher value corresponds to more robust bone regeneration, accompanied by expanded CD31- and osteocalcin (OCN)-positive areas, demonstrating coordinated vascularized and innervated bone regeneration [68]. Hou *et al.* mixed recombinant human type I collagen with natural bone composite inorganic salts at a ratio of 3:7, and compounded with 10% silk methacrylate to prepare bioinks, which were loaded with BMSCs to construct organoids. After 6 weeks of implantation into rabbit femoral defects, immunofluorescence showed that CGRP-positive nerve fibers and CD31-positive blood vessels formed a network in the defect area. The expressions of osteogenic genes (RUNX2, COL1) and neural markers were upregulated, achieving rapid neuralized and vascularized repair [69].

A major frontier in neuromuscular regenerative research is the assembly of cortical, spinal, and muscle organoids into cortico-motor assembloids, representing a landmark advance that enables reconstruction of the complete sensorimotor circuit. Rabies virus tracing confirmed projections from cortical neurons to the spinal cord, and optical stimulation of the cortex reliably triggered muscle fiber contractions. Remarkably, the assembloid system remained stable in culture for up to 10 weeks, providing a robust platform for modeling neuromuscular function and evaluating therapeutic strategies [70]. In parallel, the principles of neuralization have been applied to peripheral and planar organs such as the skin. Human iPSC-derived neuralized skin organoids could develop organized neural networks, with ISL1- and TUJ1- expressing neurons extending axons interwoven between hair follicles. Upon engraftment into mouse dorsal skin, 55% of grafts produced 2–5 mm hair, exhibiting proper orientation, integration with host epidermis, formation of cornified layers and Rete-ridge-like structures, and ingrowth of CD49F-positive vasculature. These results highlight that neuralized skin organoids can generate functional, innervated sensory structures and effectively restore planar skin at wound sites [50].

Brain injury and central nervous system (CNS) damage represent some of the most direct contexts in which neuralized organoids demonstrate regenerative potential. For instance, the developmental stage of cerebral organoids affects corticospinal reconstruction, and 10-week-old organoids exhibit enhanced axonal integration after brain injury and can extend axons in primate brains, highlighting their potential for cell-replacement therapy in cortical injury and stroke [71]. In a stroke model, hPSC-derived cerebral organoids implanted at the border of the infarct core and peri-infarct zone survived for months, differentiated into specific neuronal subtypes, reconstructed infarcted tissue, projected axons to distal brain regions, and functionally integrated into host neural circuits, ultimately restoring sensorimotor functions [72]. Similarly, human iPSC-derived midbrain organoids (hMOs) can mature into A9-type dopaminergic neurons following transplantation into the striatum of Parkinson’s disease (PD) mice. The grafted hMOs survive, extend axons, integrate with host neurons, glia, and vasculature, and are capable of reversing motor deficits without tumor formation or excessive graft growth, supporting their safety and therapeutic applicability for PD [73]. Beyond pluripotent stem cell-derived organoids, human astrocytes can also be directly reprogrammed into early neuroectodermal cells that give rise to spinal-cord organoids containing functional dorsal- and ventral-specific neurons. When transplanted into mice with complete spinal-cord injury, these organoids differentiate into spinal neurons, migrate, and form synapses with host neurons, demonstrating robust integration and repair potential for CNS injuries [74]. These studies highlight the broad regenerative potential of neuralized organoids across multiple tissues and organ systems, showing that neuralization enables the formation of intrinsic neural networks, which can subsequently achieve functional innervation and integration with host vasculature to restore motor and sensory functions in preclinical models.

Emerging evidence suggests that neural integration extends beyond motor or sensory restoration, functioning as a master regulator of both vascular and immune dynamics. Neurotrophic factors, including SC–derived exosomal signals and CGRP, can directly stimulate endothelial proliferation and angiogenesis, establishing a bidirectional neurovascular feedback loop [61, 68]. Neural signals may also modulate local immune behavior indirectly, for example by influencing macrophage activation states or cytokine profiles [75, 76]. Consequently, innervation represents not merely an additive feature but a regulatory layer that coordinates vascular support with immune readiness. Achieving durable regenerative outcomes therefore requires integrating immune competence as the third pillar of this system-level network.

### Immune cells as key regulators of tissue repair

#### The problem of an “immunologically naïve” organoid

Despite significant advances in generating vascularized and innervated organoids, most current organoids remain immunologically naive. The absence of immune components in organoid systems contributes to their incomplete maturation, lack of homeostatic stability, and vulnerability to environmental stressors or transplantation failure [77, 78]. Thus, achieving immunological competence is increasingly recognized as the next critical step toward building fully functionally organoids. Indeed, immune cells are not only the indispensable guardians of pathogen invasion but also active architects of tissue regeneration [79, 80]. This dual role of immune cells, particularly in coordinating the transition from inflammation to repair, is well-established across various regenerative contexts [81, 82]. Through regulation of hemostasis, inflammation and regenerative programs, macrophages, dendritic cells and T cell subsets control vascular growth, extracellular matrix dynamics and lineage decisions across diverse tissues, ultimately determining whether an injury resolves with regeneration or persists as chronic damage [83–85]. Yet, due to this inherent functional complexity, most *in vitro* systems do not faithfully mimic the temporal transition from inflammation to repair observed various tissues such as skin, liver, lung, and skeletal muscle without the immunoregulatory component [86, 87]. Integrating immune functionality into organoid platforms thus represents a pivotal frontier in regenerative medicine, offering the potential to achieve more mature, resilient, and therapeutically relevant tissue models [88–90].

#### Methodological approaches to establish immune control

**Figure 4:**
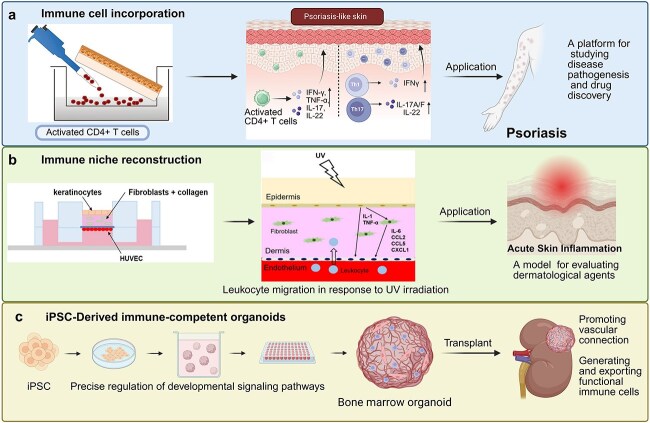
Representative approaches for immune functionalization. (**a**) Immune cell incorporation: activated human CD4-positive T cells were co-cultured with an engineered skin construct. T cell-derived cytokines induced a psoriasis-like phenotype in keratinocytes, establishing a model for pathogenesis study and drug screening. Reproduced with permission [91]. Copyright 2014, NATURE PUBLISHING GROUP. (**b**) Immune niche reconstruction: a 3D skin-on-a-chip platform was constructed, comprising keratinocytes, fibroblasts, and a collagen matrix, integrated with a vessel-like structure formed by HUVECs. Upon UV irradiation, introduced HL-60 leukocytes migrated from the vessel into the skin compartment, recapitulating acute inflammatory cell infiltration. Reproduced with permission [94]. Copyright 2020, JOHN WILEY & SONS, INC. (**c**) iPSC-derived immune-competent organoids: iPSC-derived BMOs were generated through precise regulation of developmental signaling pathways. Following *in vivo* transplantation, these assembloids established vascular connections with the host, achieved functional integration, and generated/exported functional immune cells. *iPSCs* induced pluripotent stem cells, *HUVECs* human umbilical vein endothelial cells, *IL* interleukin, *TNF-α* tumor necrosis factor-α

Immune cell incorporation: a foundational strategy involves co-culturing organoids with exogenous immune cells to reconstruct tissue-immune crosstalk. For example, activated CD4^+^ T cells have been introduced into engineered skin equivalents, migrating within a 3D microenvironment to induce keratinocyte inflammatory responses reminiscent of psoriasis (Fig. 4a) [91]. Similar strategies have been employed in lung organoids, where peripheral blood-derived T cells and macrophages were exogenously introduced during the early stage of organoid morphogenesis. The added immune cells interact with epithelial and stromal cells, guiding morphogenesis, remodeling extracellular matrices, and modulating differentiation trajectories [92]. During the differentiation of adipose organoids, the stromal vascular fraction (SVF) containing endogenous immune cells was incorporated through direct co-culture, which successfully retained CD45-positive immune cells (predominantly macrophages). Upon lipopolysaccharide (LPS) stimulation, these organoids secreted inflammatory cytokines such as IL-6 and tumor necrosis factor-α (TNF-α) [93]. In brain organoids, co-culture of iPSC-derived primitive neural progenitor cells and primitive macrophage progenitors at the initiation of organoid construction enabled coordinated differentiation and maturation, generating functional microglia with controllable cell ratios [77]. These early attempts have demonstrated clearly that immune cells within organoids are not inert occupants, they play active roles in shaping tissue behavior, function, and remodeling. However, immune cells introduced exogenously often exhibit immature phenotypes, limited plasticity, and poor long-term survival, making them best suited for studying acute rather than chronic immune dynamics. Their integration also depends heavily on the ability to support immune niches, which are often underdeveloped.

Immune niche reconstruction: Tissue engineering and microfabrication have laid essential groundwork for generating immune-integrated organoids by mimicking the architecture, stroma and microenvironment of the authentic tissue which provides a better premise for immune integration. A recent study constructed a 3D skin model with epidermis, dermis, and a perfusable vascular endothelial layer on a microfluidic chip to mimic immune cell intravasation from blood vessels into surrounding tissue. Pre-differentiated HL-60 neutrophil precursors introduced into the vascular channels successfully migrated into and integrated with the artificial skin tissue (Fig. 4b) [94]. Moreover, mast cells can also be integrated into the dermis by co-culturing them with 3D skin organoid with keratinocytes on a fibroblast-containing collagen matrix. Even in the absence of additional cytokine support, these cells stably persisted in the model, retained degranulation function, and established functional crosstalk with both stromal and epithelial cells [95]. Yet, one major disadvantage of such systems was that they still relied on exogeneous immune cells that lacked developmental synchrony with host tissues.

iPSC-derived immune-competent organoids: to fundamentally resolve challenges such as developmental asynchrony and immunological incompatibility, recent strategies in organoid research have shifted from externally engineered immune addition to the intrinsic, co-emergent development of immune cells within organoids derived from iPSCs. Therefore, this approach ensures ontogenetic synchrony and genetic uniformity between immune and parenchymal lineages, and it fosters more stable, long-term functional integration [96]. A breakthrough study established a serum- and feeder-free 3D inductive system that differentiated human iPSCs into bone marrow organoids (BMOs) within 21 days. Stepwise activation of FGF, BMP, and VEGF signaling sequentially guided the formation of vascular networks, mesenchymal stromal cells, and multi-lineage hematopoietic cells, and autonomously produced neutrophils, monocytes, megakaryocytes, and lymphoid progenitors without exogenous immune input, recapitulating the molecular cues underlying human hematopoietic niche formation. This work enhances the authenticity of organoids in simulating the human immune microenvironment and supports the development of functional hematopoietic tissues and disease models *in vitro* (Fig. 4c) [97]. Importantly, they represented a transition from transient, reductionist models toward stable, physiologically relevant immune niches capable of modeling hematopoiesis, infection, inflammation, and tissue repair. Similar advances were made in kidney organoids, where co-culture with peripheral blood mononuclear cells allowed T cells and macrophages to infiltrate the parenchyma and engage in bidirectional signaling with epithelial cells, leading to altered tissue patterning and morphogenesis [98]. These interactions further reinforced the idea that immune integration is not a peripheral accessory, but an emergent and essential property of mature organoid systems.

#### Regenerative medicine applications and evidence

Recent advances in immune-organoid integration have revealed that immune cells not only actively shape organoid development but also recapitulate the authentic signaling networks between immune cells and their surrounding tissues leading to markedly enhanced regenerative capacity. While not all systems have been used in the context of tissue repair modeling or regenerative medicine in the clinic, they have shown great potential in recapitulating the underlying biological characteristics in development and disease alike.

In human skin equivalents, co-culturing with CD4^+^ T cells induce hallmark features of psoriatic inflammation, s including activation of T cells and upregulation of IFN-γ, TNF-α, IL-17, and IL-22, which in turn downregulate FLG (filaggrin) and disrupt IVL (involucrin) expression in keratinocytes. This cytokine-mediated feedback drives aberrant differentiation and establishes a self-sustaining inflammatory microenvironment, faithfully modeling the immune-epithelial crosstalk underlying psoriatic pathology (Fig. 4a) [91]. In lung organoids, incorporation of tissue-resident macrophages (TR-Macs) reprograms epithelial gene expression, suppressing proliferation-related genes while activating maturation-associated genes such as Neat1, Cyp2f2, and Ces1d. During simulated influenza infection, epithelial signals activate TR-Macs to secrete pro-inflammatory cytokines, amplifying innate immune defense and accelerating tissue repair, thus revealing the molecular circuitry of immune-epithelial communication [92]. Similarly, in bone organoids, the introduction of CD14^+^ monocytes enabled osteoclast differentiation and active matrix resorption, successfully recapitulating the dynamic equilibrium of bone remodeling observed *in vivo* [99].

**Table 2:** Comparative overview of co-culture systems, genetic engineering, biomaterials, and microengineering approaches for integrated organoids

	Advantages	Limitations	Refs.
Co-culture systems			
Vascular	Self-assembly enables formation of vascular networks with tight junctions and basement membranes Supports co-differentiation under endogenous cues	Networks are highly random and difficult to control; Require multiple cell types	[23–25, 38]
Neural	Enables spontaneous formation of specialized cell–cell connections (e.g. NMJs) Supports spontaneous formation of specialized cell–cell connections (e.g. NMJs)	System complexity and limited controllability Limited long-term stability	[66, 67]
Immune	Replicates tissue architecture and intercellular crosstalk Enables real-time observation of immune cell dynamics	Models inflammation, but cannot fully recapitulate typical disease features Frequently exhibits an immature immune state	[91, 92]
Genetic engineering			
Vascular	Activating transcription factors induces progenitors to co-differentiate into endothelial and mural cells Endothelial cells secrete signals that guide tissue patterning and organoid maturation	Requires precise temporal control of gene expression May not fully capture native vascular complexity without extra microenvironmental cues	[26, 27]
Neural	Recapitulates functional neuro-tissue organization through self-assembly	Networks are complex and time-consuming to construct Neural components often exhibit immature state	[50, 63]
Immune	Reconstructs complex multi-cellular microenvironments with a diverse array of cell types Supports the emergence of self-organized tissue architecture Enables the induction of functional immune responses	Limited tissue maturity affects transcriptomic and immune responses Absence of vascular flow restricts immune cell trafficking Reduced immune diversity does not capture natural tissue complexity	[96–98]
Biomaterials			
Vascular	Biomaterials mimic extracellular matrix and provide mechanical support for vascular networks Biochemical cues (e.g. RGD motifs) trigger angiogenic signaling	Limited long-term stability Requires compatibility with specific cell types	[42, 44]
Neural	Provides physical guidance and support for neurite outgrowth through biomaterial scaffolds Enables formation of multi-tissue interactive niches (e.g. neuro-osteogenic niches)	Incomplete replication of native neural microenvironment Random spatial distribution of cells within scaffolds Dependence on exogenous growth factors for long-term survival Limited nutrient supply in thick scaffold centers	[60, 64]
Microengineering			
Vascular	Microfluidics/3D printing allow precise vascular patterning (e.g. gradient control) Dynamic flow promotes maturation Enables real-time monitoring	High cost and technical complexity Limited scalability for clinical applications	[45, 46, 54]
Neural	Enables precise spatial organization of neural components through 3D printing Allows real-time monitoring and control of neural activity	High construction complexity and cost Functional maturity below adult tissue level	[60, 62, 64, 65]
Immune	Supports spatiotemporally precise manipulation of cell populations Enables the induction of functional immune responses	Lacks adult-level physiological maturity Requires high resource investment and specialized maintenance	[94]

These interactions can be even better recapitulated in iPSC-derived organoids, which generate autologous immune compartments alongside parenchymal lineages. In iPSC-derived BMOs, serum-free self-organizing systems activate Notch pathway signaling in arterial-like endothelial cells, which drives the transition of hematopoietic progenitors toward lymphoid commitment through IL-7R and FLT3 expression. Neutrophil maturation proceeds through the regulated expression of CD11b, CD101, and CD16, accompanied by effector molecules MPO and S100A8/A9. In VPS45-deficient BMO models, increased Annexin V expression in mature neutrophils reveals the abnormal apoptotic pathway that causes neutropenia in patients (Fig. 4c) [97]. In kidney organoids, the incorporation of immune cells triggered bidirectional signaling that altered epithelial patterning and morphogenesis [98]. In adipose organoids, tissue-resident macrophages and mast cells not only retain their inflammatory responsiveness but also inhibit the insulin signaling pathway via pro-inflammatory factors such as TNF-α and IL-6. These immune cells secreted inosine to enhance the thermogenic activity of brown and beige adipocytes and regulate extracellular matrix remodeling [93]. These systems illustrate that immune cells are not mere add-ons but dynamic regulators of organoid architecture, tissue patterning, and overall functional integration. Nevertheless, whether immune properties established *in vitro* can be retained post-transplantation, and how host-graft immune interactions may reprogram these characteristics, remain open questions. Current models still lack adaptive immune features, organized lymphoid structures, and systemic surveillance, all of which must be addressed to realize durable, functional immune-competent organoid therapies.

Taken together, immune integration completes the functional interplay among vascular, neuronal, and immune systems in organoid-based regeneration. Immune cells regulate angiogenesis and support neural regeneration through growth factors and cytokines such as VEGF, PDGF, and IGF-1 [81]. In turn, vascular and neural signals modulate immune cell recruitment, activation, and resolution [100–102]. Through these reciprocal interactions, the three systems collectively achieve functional synergy, enabling emergent regenerative behaviors that would not occur in isolation.

### The road to clinical translation

Recent progress in vascularization, innervation, and immune integration has propelled organoid research from structural mimicry to functional regeneration, with a comparative overview of each method presented in Table 2. However, clinical translation remains an ongoing challenge. Current advances represent crucial but still preliminary steps, as most organoids exhibit only partial functionality and fall short of meeting the diverse regenerative demands across injury types, such as ischemic, inflammatory, traumatic, and degenerative lesions [103]. For instance, vascularized and innervated constructs suitable for bone or muscle regeneration may not recapitulate the unique regenerative microenvironment required for skin, liver, or nervous tissue repair. Achieving system-level functionality requires tailoring organoid design to specific tissue contexts, validating underlying regenerative mechanisms *in vivo*, and ensuring coordinated restoration of multiple tissue compartments.

As the system most commonly involved in wound healing, skin organoids represent a particularly relevant model for regenerative applications, integrating vascular, neural, and immune components critical for tissue repair. Despite the advantages of current skin organoid models, PSC-derived skin organoids (SKOs), while capable of generating skin and neural components from ectodermal lineages, remain limited by the absence of functional blood vessels and immune cells from the mesoderm [50]. Recent studies show that hiPSC-derived VOs can be incorporated into SKOs to generate fully vascularized skin organoids, which recapitulate key vascular-immune interactions within the dermis and provide a physiologically relevant platform for regenerative applications [104]. Compared with SKOs alone, VOs provide endothelial, perivascular, and stromal components that better mimic the dermal niche, supporting potential regenerative therapies for ischemic, inflammatory, and traumatic skin injuries. Transplantation outcomes for these injury types still require further investigation to advance their translational potential in regenerative medicine.

Beyond biological optimization, practical challenges in manufacturing and quality control remain critical barriers [105]. While single-lineage organoids already face issues of reproducibility, maturation, and functional integration, the production of complex, multi-lineage organoids further amplify these demands. Translating laboratory-scale fabrication to clinical-grade biomanufacturing requires compliance with good manufacturing practice standards to ensure genomic stability, product consistency, and long-term safety [106]. Success will depend on scalable bioprocessing systems, precise spatiotemporal control of multi-lineage differentiation, integrated vascular, neural, and immune modules, standardized quality assessment criteria, and validated cryopreservation protocols, that collectively guarantee reproducibility across batches and manufacturing sites.

Stem cell-based organoids bioengineering offers translational pathway toward fully functional, system-level tissue constructs. Both patient-derived ASCs and iPSCs can mitigate immune rejection, enabling the reconstruction of integrated vascular, neural, and immune networks rather than isolated cell populations [107, 108]. Early clinical applications will likely target accessible tissues such as skin and muscle, where functional organoid patches could simultaneously promote wound closure, reinnervation, and revascularization. By coordinating multiple regenerative modules, these organoids establish a foundation for progressively rebuilding more complex internal organs and fully functional biological systems.

## Conclusions

The evolution of organoid technology signifies a paradigm shift from simplified *in vitro* models to living, functional therapeutics capable of system-level physiological integration and repair. Vascularization, innervation, and immune competence together form the triad of regenerative completeness, enabling organoids to transition from self-contained mini-tissues to dynamic participants in host biology. Realizing this vision will require deep interdisciplinary collaboration that unites stem cell biologists, tissue engineers, immunologists, and clinicians to translate bioengineered complexity into clinical efficacy. Through such collective effort, organoid science is poised to redefine the boundaries of regenerative medicine and deliver tangible benefits to patients.

## Data Availability

Not applicable.

## Abbreviations

3D: three-dimensional, Aβ: amyloid-beta, AChR/AChRs: acetylcholine receptor(s), AD: Alzheimer’s disease, ANGPT1: angiopoietin-1, Annexin V: annexin A5, ASCs: adult stem cells, BDNF: brain-derived neurotrophic factor, BMOs: bone marrow organoids, BMP: bone morphogenetic protein, BMSCs: bone marrow mesenchymal stem cells, BV: bone volume, BV/TV: bone volume/total volume, CD11b: cluster of differentiation 11b, CD16: cluster of differentiation 16, CD31: cluster of differentiation 31, CD101: cluster of differentiation 101, CGRP: calcitonin gene-related peptide, CNS: central nervous system, CS: calcium silicate, DLP: digital light processing, EC/ECs: endothelial cell(s), ECM: extracellular matrix, ENS: enteric nervous system, ERK: extracellular signal-regulated kinase, ETV2: ETS variant transcription factor 2, FGF/FGF2: fibroblast growth factor, FLG: filaggrin, FLT3: Fms-related receptor tyrosine kinase 3, FLT4: Fms-related receptor tyrosine kinase 4, GDNF: glial cell-derived neurotrophic factor, GelMA: gelatin methacryloyl, GMP: good manufacturing practice, hMOs: human iPSC-derived midbrain organoids, hPSC: human pluripotent stem cell, HUVECs: human umbilical vein endothelial cells, IFN-γ: interferon-gamma, IGF: insulin-like growth factor, IL-6: interleukin-6, IL-7R: interleukin-7 receptor, IL-8: interleukin-8, IL-17: interleukin-17, IL-22: interleukin-22, IL6ST: interleukin-6 signal transducer, iPSCs: induced pluripotent stem cells, ISL1: ISL LIM homeobox 1, IVL: involucrin, LPS: lipopolysaccharide, MAPK: mitogen-activated protein kinase, MPO: myeloperoxidase, MPS: microphysiological system, MuSK: muscle-specific kinase, NGF: nerve growth factor, NKX3.1: NK3 homeobox 1, NMJs: neuromuscular junctions, NMOs: neuromuscular organoids, Notch3: Notch receptor 3, NRG1: neuregulin-1, OCN: osteocalcin, PCNA: proliferating cell nuclear antigen, PD: Parkinson’s disease, PDGF/PDGFB: platelet-derived growth factor, PI3K-Akt: phosphoinositide 3-kinase/Akt pathway, RGD: arginine-glycine-aspartate motif, RUNX2: runt-related transcription factor 2, SC-exos: Schwann cell-derived exosomes, SCs: Schwann cells, SilMA: silk methacryloyl, SKOs: skin organoids, Smad: SMAD family protein, S100A8/A9: S100 calcium-binding protein A8/A9, SVF: stromal vascular fraction, TGF-β: transforming growth factor-beta, TNF-α: tumor necrosis factor-α, TR-Macs: tissue-resident macrophages, TUJ1: beta III-tubulin, TV: total volume, VE-cadherin: vascular endothelial cadherin, VEGF: vascular endothelial growth factor, VOs: vascular organoids, VSKOs: vascularized skin organoids, Wnt: Wingless-related integration site signaling pathway, YAP/TAZ: Yes-associated protein/transcriptional coactivator with PDZ-binding motif, α5β1: integrin alpha-5 beta-1, αvβ3: integrin alpha-v beta-3, β-TCP: beta-tricalcium phosphate
